# Physical Education in Relation to Mental Development in School-Life

**Published:** 1890-08

**Authors:** Thomas More Madden

**Affiliations:** Physician to the Hospital for Sick Children, Dublin; Obstetric Physician and Gynæcologist Mater Misericordiæ Hospital; Examiner Conjoint Board Royal College of Surgeons and Apothecaries’ Hall, Ireland; Consultant National Lying-in-Hospital; ex-President Obstetric Sections of the Royal Academy of Medicine, Ireland, and of the British Medical Association; Formerly Vice-President British Gynæcological Society; M. D. Honoris Causa Texas Medical College, etc.


### PHYSICAL EDUCATION IN RELATION TO MENTAL DEVELOPMENT IN SCHOOL-LIFE.

BY THOMAS MORE MADDEN, M. D., F. R. C. S. ED.,

Physician to the Hospital for Sick Children, Dublin; Obstetric Physician and Gynæcologist Mater Misericordiæ Hospital; Examiner Conjoint Board Royal College of Surgeons and Apothecaries' Hall, Ireland; Consultant National Lying-in-Hospital; ex-President Obstetric Sections of the Royal Academy of Medicine, Ireland, and of the British Medical Association; Formerly Vice-President British Gynæcological Society; M. D. *Honoris Causa* Texas Medical College, etc.*

The respective claims of physical and mental training, and the evils arising from neglect or abuse of either are obviously questions of the highest medical as well as social interest. This neglect now presents itself in two different aspects. On the one hand, the children of the poor in England are compulsorily subjected at an absurdly early age, to a forcing and injurious system of mental cultivation. Whilst on the other hand, in the case of those of a better social position, the physical powers are not uncommonly overstrained, at the expense of the mental faculties. Of these errors, the former is the most important, and to its operation is, I believe, largely ascribable the apparent diminution of physical stamina observable in too many of the youth of the present day as compared with the physically more robust, if intellectually less cultured generation of the pre-educational period. Looking at the overtasked and anæmic little children now chained to the desk by the School Boards, we might be tempted to believe

> " 'Twas not the sires of such as these
> Who dared the elements and pathless seas;
> But beings of another mould—
> Rough, hardy, vigorous, manly, bold!"

At the present time, a large part of the first ten years of life, which should be primarily devoted to physical and moral training, is given up to the development of the mental powers: the child, when a mere infant, being compelled to attend some school, where the immature brain is forced into abnormal and disastrous activity. On its return home, jaded in mind and

*Abstract of a paper for Section Diseases of Children—British Medical Association, Birmingham, July, 1890.

body, to prepare for next day's task, such a child is necessarily unfit for the enjoyment of the physical exercise which is essential for its bodily development and health, or for its still more important elementary training of the affections and moral faculties and instilment of religious principles, which are better acquirable from home teachings than from any School Board system. We are all, of course, agreed as to the duty of properly educating children so as to fit them mentally and bodily for the increasing requirements and competition of modern life. But as to the extent to which the former should be carried and the latter neglected in early childhood, there is unfortunately a great discrepancy between the rulers of the Educational Department and the views of those who have to deal in disease with the consequences of the violation of the laws of nature. And hence, whilst little children are thereby overworked into disease or death, the physician must still raise his protesting voice, albeit it would apparently seem unheeded.

During the first eight or ten years of child life, the amount of mental cultivation which a child's brain is capable of receiving with permanent advantage is much less than is commonly believed. No greater physiological mistake is possible than that of attempting any considerable degree of such culture until the sufficient development of the physical stamina and moral faculties is accomplished. The organ of the mind is as much a part of the body as the hand, and ere either can function properly, its vital force must be fostered and maintained by nutrition and developed by physical exercise. A large proportion of those who come within the provisions of the Elementary Education code are semi-starved children of the poorest class, who, when thus debilitated by privation, are necessarily as much incapacitated for any mental strain as for the accomplishment of any herculean feat of physical strength: it being not less inhuman, injudicious, and impolitic to expect the former than it would be the latter from those so circumstanced.

If the State, for reasons of public policy, determines that all children shall be compulsorily educated from their earliest years, it should certainly afford the means by which this may be least injuriously and most effectually carried out, by providing food and physical training as well as mental education for every pauper child attending an Elementary school.

Among the results of over-pressure in such schools under the Boards referred to, are brain diseases in all forms—viz., cephalitis, cerebritis and meningitis, as well as headache, sleeplessness, neuroses of every kind, and other evidences of cerebro-nervous disorders. On no other ground can the increasing prevalence of these affections amongst the little victims of the Educational Department be accounted for or explained, than by ascribing them to the new factors "brain excitement" and "over-pressure," which, in the case of young children, are now too commonly disastrously associated with the process of misdirected education and neglected physical training.

In connection with the physical management of childhood, I may add a few words on the abuse of alcoholic stimulants. The evils resulting from the abuse of alcohol were never so prevalent as at present, and are traceable in the diseases of youth as well as in those of adult existence. The results of this acquired or inherited alcoholism are brought under clinical observation in the form of cerebral, gastric and hepatic disorders, and especially cirrhosis of the liver, which as well as the protean forms of cerebro-spinal disease, and the various neuroses so frequently noticed in hospitals for children, and to which I have elsewhere directed attention. In the majority of these cases of juvenile alcoholism that have come under my care in the Children's Hospital, Dublin, this tendency appears inherited and most marked in those whose mothers were inebriates—intemperance in women also bearing in other ways on the diseases treated in hospitals for children, where its effects are strikingly evinced by the moral and physical deterioration of the offspring of the drunken and by their special predisposition to strumous, tubercular and other constitutional taints.

Under no circumstances should alcoholic stimulants be given to children, save in the guise and defined doses of other remedial agents—my experience in hospital and private practice, at home and abroad, having amply confirmed the view expressed in a work of mine published many years since, viz., that it is physiologically wrong, as well as morally unjustifiable, ever to allow a healthy child to taste alcohol in any form.

